# BGX: a Bioconductor package for the Bayesian integrated analysis of Affymetrix GeneChips

**DOI:** 10.1186/1471-2105-8-439

**Published:** 2007-11-12

**Authors:** Ernest Turro, Natalia Bochkina, Anne-Mette K Hein, Sylvia Richardson

**Affiliations:** 1 Centre for Biostatistics, Imperial College London, UK; 2 Department of Mathematics, University of Edinburgh, UK; 3 Molecular Diagnostic Laboratory, Skejby Hospital, Aarhus University, Denmark

## Abstract

**Background:**

Affymetrix 3' GeneChip microarrays are widely used to profile the expression of thousands of genes simultaneously. They differ from many other microarray types in that GeneChips are hybridised using a single labelled extract and because they contain multiple 'match' and 'mismatch' sequences for each transcript. Most algorithms extract the signal from GeneChip experiments in a sequence of separate steps, including background correction and normalisation, which inhibits the simultaneous use of all available information. They principally provide a point estimate of gene expression and, in contrast to BGX, do not fully integrate the uncertainty arising from potentially heterogeneous responses of the probes.

**Results:**

BGX is a new Bioconductor R package that implements an integrated Bayesian approach to the analysis of 3' GeneChip data. The software takes into account additive and multiplicative error, non-specific hybridisation and replicate summarisation in the spirit of the model outlined in [1]. It also provides a posterior distribution for the expression of each gene. Moreover, BGX can take into account probe affinity effects from probe sequence information where available. The package employs a novel adaptive Markov chain Monte Carlo (MCMC) algorithm that raises considerably the efficiency with which the posterior distributions are sampled from. Finally, BGX incorporates various ways to analyse the results, such as ranking genes by expression level as well as statistically based methods for estimating the amount of up and down regulated genes between two conditions.

**Conclusion:**

BGX performs well relative to other widely used methods at estimating expression levels and fold changes. It has the advantage that it provides a statistically sound measure of uncertainty for its estimates. BGX includes various analysis functions to visualise and exploit the rich output that is produced by the Bayesian model.

## Background

Oligonucleotide microarrays allow biomedical researchers to estimate the expression of thousands of genes simultaneously through their mRNA transcripts. A labelled, fragmented version of the RNA may be hybridised onto an array containing hundreds of thousands of complementary oligonucleotides and then scanned. Affymetrix 3' GeneChip arrays represent genes by sets of probe pairs, each of which consists of an oligonucleotide of length 25 which matches a corresponding RNA subsequence perfectly (PM) and an identical probe with an inverted oligonucleotide on position 13 (MM) that is intended to measure non-specific hybridisation.

The BGX model [1] is an integrated approach to the analysis of GeneChip microarrays in which correction for non-specific hybridisation and gene expression level estimation are performed simultaneously. Posterior distributions of parameters in the model may be obtained numerically. Based on these distributions, a powerful method for detecting differential expression has been developed [2].

The probes on Affymetrix GeneChips have been found to exhibit varying propensities to "shine" according to the base composition of their sequences [3] and methods for estimating expression levels from GeneChips that incorporate probe affinity effects have shown demonstrable advances over methods in which these effects are ignored (see, e.g. [4]). We present a new Bioconductor [5] package that implements the BGX model, includes an extension to incorporate probe affinity effects, employs novel algorithmic techniques to sample effectively from posterior distributions, and provides various analysis and plotting functions.

### Implementation

#### Basic model

BGX [1] explicitly models probe intensities as arising partly from specific hybridisation, *S *(the signal), and partly from non-specific hybridisation, *H*, with only a fraction, *φ*, of the signal occuring at a PM probe also occurring at the corresponding MM probe. The *S*s and *H*s are gene (*g*), probe (*j*), condition (*c*) and replicate (*r*) specific, and the intensities are assumed to be affected by an additive array-specific noise:

PMgjcr~N(Sgjcr+Hgjcr,τcr2),
 MathType@MTEF@5@5@+=feaafiart1ev1aaatCvAUfKttLearuWrP9MDH5MBPbIqV92AaeXatLxBI9gBaebbnrfifHhDYfgasaacPC6xNi=xI8qiVKYPFjYdHaVhbbf9v8qqaqFr0xc9vqFj0dXdbba91qpepeI8k8fiI+fsY=rqGqVepae9pg0db9vqaiVgFr0xfr=xfr=xc9adbaqaaeGacaGaaiaabeqaaeqabiWaaaGcbaGaeeiuaaLaeeyta00aaSbaaSqaaiabdEgaNjabdQgaQjabdogaJjabdkhaYbqabaGccqGG+bGFcqWGobGtcqGGOaakcqWGtbWudaWgaaWcbaGaem4zaCMaemOAaOMaem4yamMaemOCaihabeaakiabgUcaRiabdIeainaaBaaaleaacqWGNbWzcqWGQbGAcqWGJbWycqWGYbGCaeqaaOGaeiilaWccciGae8hXdq3aa0baaSqaaiabdogaJjabdkhaYbqaaiabikdaYaaakiabcMcaPiabcYcaSaaa@4E51@

MMgjcr~N(φSgjcr+Hgjcr,τcr2).
 MathType@MTEF@5@5@+=feaafiart1ev1aaatCvAUfKttLearuWrP9MDH5MBPbIqV92AaeXatLxBI9gBaebbnrfifHhDYfgasaacPC6xNi=xI8qiVKYPFjYdHaVhbbf9v8qqaqFr0xc9vqFj0dXdbba91qpepeI8k8fiI+fsY=rqGqVepae9pg0db9vqaiVgFr0xfr=xfr=xc9adbaqaaeGacaGaaiaabeqaaeqabiWaaaGcbaGaeeyta0Kaeeyta00aaSbaaSqaaiabdEgaNjabdQgaQjabdogaJjabdkhaYbqabaGccqGG+bGFcqWGobGtcqGGOaakiiGacqWFgpGzcqWGtbWudaWgaaWcbaGaem4zaCMaemOAaOMaem4yamMaemOCaihabeaakiabgUcaRiabdIeainaaBaaaleaacqWGNbWzcqWGQbGAcqWGJbWycqWGYbGCaeqaaOGaeiilaWIae8hXdq3aa0baaSqaaiabdogaJjabdkhaYbqaaiabikdaYaaakiabcMcaPiabc6caUaaa@5003@

The log-transformed signal parameter, log(*S*_*gjcr *_+ 1), is assumed to follow a gene and condition specific distribution, while the log-transformed non-specific hybridisation term, log(*H*_*gjcr *_+ 1), is assumed to arise from an array-specific distribution:

log⁡(Sgjcr+1)~TN(μgc,σgc2),
 MathType@MTEF@5@5@+=feaafiart1ev1aaatCvAUfKttLearuWrP9MDH5MBPbIqV92AaeXatLxBI9gBaebbnrfifHhDYfgasaacPC6xNi=xI8qiVKYPFjYdHaVhbbf9v8qqaqFr0xc9vqFj0dXdbba91qpepeI8k8fiI+fsY=rqGqVepae9pg0db9vqaiVgFr0xfr=xfr=xc9adbaqaaeGacaGaaiaabeqaaeqabiWaaaGcbaGagiiBaWMaei4Ba8Maei4zaCMaeiikaGIaem4uam1aaSbaaSqaaiabdEgaNjabdQgaQjabdogaJjabdkhaYbqabaGccqGHRaWkcqaIXaqmcqGGPaqkcqGG+bGFcqWGubavcqWGobGtcqGGOaakiiGacqWF8oqBdaWgaaWcbaGaem4zaCMaem4yamgabeaakiabcYcaSiab=n8aZnaaDaaaleaacqWGNbWzcqWGJbWyaeaacqaIYaGmaaGccqGGPaqkcqGGSaalaaa@4C0A@

log⁡(Hgjcr+1)~TN(λcr,ηcr2),
 MathType@MTEF@5@5@+=feaafiart1ev1aaatCvAUfKttLearuWrP9MDH5MBPbIqV92AaeXatLxBI9gBaebbnrfifHhDYfgasaacPC6xNi=xI8qiVKYPFjYdHaVhbbf9v8qqaqFr0xc9vqFj0dXdbba91qpepeI8k8fiI+fsY=rqGqVepae9pg0db9vqaiVgFr0xfr=xfr=xc9adbaqaaeGacaGaaiaabeqaaeqabiWaaaGcbaGagiiBaWMaei4Ba8Maei4zaCMaeiikaGIaemisaG0aaSbaaSqaaiabdEgaNjabdQgaQjabdogaJjabdkhaYbqabaGccqGHRaWkcqaIXaqmcqGGPaqkcqGG+bGFcqWGubavcqWGobGtcqGGOaakiiGacqWF7oaBdaWgaaWcbaGaem4yamMaemOCaihabeaakiabcYcaSiab=D7aOnaaDaaaleaacqWGJbWycqWGYbGCaeaacqaIYaGmaaGccqGGPaqkcqGGSaalaaa@4C07@

where *TN *denotes the truncated normal distribution, truncated to the positive axis. The central parameter of equation (3), *μ*_*gc*_, acts as the BGX expression measure, and equations (1) to (4) represent the basic BGX model.

The core of the model is implemented in the C++ programming language for efficiency and uses MCMC to sample from the full posterior distributions of each parameter. Parameters are estimated using Gibbs sampling where possible (*φ *and *τ*) and a Random Walk Metropolis-Hastings algorithm elsewhere (*S*, *H*, *μ*, *σ*, *λ *and *η*). Three C++ class templates are used to instantiate zero, one and two-dimensional MCMC update objects for each parameter according to the dimensionality of the corresponding suffixes. Each of the instantiated objects is updated in sequence using references to all other necessary parameters during a burn-in period, which is discarded, and a sampling period, which is used for the posterior distributions.

#### Probe affinity extension

It has been observed that the propensity of probes to hybridise to mRNA is affected by their base composition [3]. In particular, probes with a high number of cytosine bases have a high propensity to hybridise while probes with a high number of adenine bases exhibit the opposite tendency. Moreover, the nearer the bases are to the centre of the oligonucleotide, the greater the effect. We account for this in an extension to the core model that incorporate affnity effects in the modelling of non-specific hybridisation. We categorise probes in the following way: let α be a function which, for each gene and probe pair, (*g, j*), gives the affnity category of a given probe: *α *: (*g, j*) → {1, ..., *K*} (defined below). We refine equation (4) by allowing for a category and array specific distribution of the non-specific hybridisation parameter:

log⁡(Hgjcr+1)~TN(λcrα(g,j),ηcr2).
 MathType@MTEF@5@5@+=feaafiart1ev1aaatCvAUfKttLearuWrP9MDH5MBPbIqV92AaeXatLxBI9gBaebbnrfifHhDYfgasaacPC6xNi=xI8qiVKYPFjYdHaVhbbf9v8qqaqFr0xc9vqFj0dXdbba91qpepeI8k8fiI+fsY=rqGqVepae9pg0db9vqaiVgFr0xfr=xfr=xc9adbaqaaeGacaGaaiaabeqaaeqabiWaaaGcbaGagiiBaWMaei4Ba8Maei4zaCMaeiikaGIaemisaG0aaSbaaSqaaiabdEgaNjabdQgaQjabdogaJjabdkhaYbqabaGccqGHRaWkcqaIXaqmcqGGPaqkcqGG+bGFcqWGubavcqWGobGtcqGGOaakiiGacqWF7oaBdaqhaaWcbaGaem4yamMaemOCaihabaGae8xSdeMaeiikaGIaem4zaCMaeiilaWIaemOAaOMaeiykaKcaaOGaeiilaWIae83TdG2aa0baaSqaaiabdogaJjabdkhaYbqaaiabikdaYaaakiabcMcaPiabc6caUaaa@52EC@

The extended model, which we denote GCBGX, is based on equations (1), (2), (3) and (5).

The probes on the arrays are, prior to analysis, grouped into a number of probe affinity categories. This is done by: (a) calculating the probe affinities using the *gcrma *Bioconductor package [4], (b) rounding them to the first decimal place, (c) assigning each value to a preliminary probe affinity category and (d) ensuring that the final categories contain a sufficient number of probes by collapsing small preliminary categories together. We enumerate the resulting probe affinity categories 1, ... , *K *by increasing affinity. Once categorised, the affinity-specific parameters are estimated from the data, simultaneously with all other parameters.

In some cases, Affymetrix do not directly provide the sequences for all probesets due to licensing restrictions and, consequently, there are Bioconductor probe packages that do not contain complete sequence information. For example, *hgu95aprobe *(version ≤ 1.16.2) lacks sequences for probes belonging to 172 probesets. We tackled this problem by treating *α*(*g, j*) as a random variable, taking values from 1 to *K*, with prior probability equal to the observed frequency of the categories, pk=NkN
 MathType@MTEF@5@5@+=feaafiart1ev1aaatCvAUfKttLearuWrP9MDH5MBPbIqV92AaeXatLxBI9gBaebbnrfifHhDYfgasaacPC6xNi=xH8viVGI8Gi=hEeeu0xXdbba9frFj0xb9qqpG0dXdb9aspeI8k8fiI+fsY=rqGqVepae9pg0db9vqaiVgFr0xfr=xfr=xc9adbaqaaeGacaGaaiaabeqaaeqabiWaaaGcbaGaemiCaa3aaSbaaSqaaiabdUgaRbqabaGccqGH9aqpjuaGdaWcaaqaaiabd6eaonaaBaaabaGaem4AaSgabeaaaeaacqWGobGtaaaaaa@343F@, where *N*_*k *_is the number of probes in category *k *and N=∑k=1KNk
 MathType@MTEF@5@5@+=feaafiart1ev1aaatCvAUfKttLearuWrP9MDH5MBPbIqV92AaeXatLxBI9gBaebbnrfifHhDYfgasaacPC6xNi=xH8viVGI8Gi=hEeeu0xXdbba9frFj0xb9qqpG0dXdb9aspeI8k8fiI+fsY=rqGqVepae9pg0db9vqaiVgFr0xfr=xfr=xc9adbaqaaeGacaGaaiaabeqaaeqabiWaaaGcbaGaemOta4Kaeyypa0ZaaabmaeaacqWGobGtdaWgaaWcbaGaem4AaSgabeaaaeaacqWGRbWAcqGH9aqpcqaIXaqmaeaacqWGlbWsa0GaeyyeIuoaaaa@3719@.

#### Adaptive MCMC

The full conditional distributions of *S*, *H*, *μ*, *σ*, *λ *and *η *are updated by drawing new values from a proposal distribution, typically a Random Walk (RW) Gaussian proposal centred on the current value with a chosen variance. A typical experiment consists of several hundred thousand probes, resulting in potentially millions of *S *and *H *components and tens of thousands of *μ *and *σ *components. Each component of a given parameter has a different support and consequently a different optimal RW proposal variance. Using a fixed variance for all components results in excessively low or high acceptance ratios for a large proportion of components, leading to highly autocorrelated chains.

In order to tackle this problem, we implemented the novel Adaptive Metropolis-Within-Gibbs algorithm recently proposed by Roberts and Rosenthal [6,7]. We used a unique proposal variance for each object, which adapts to its optimal value after successive batches of 50 iterations. The aim is to achieve an acceptance ratio of around 0.44, which has been shown to be optimal for one-dimensional proposals in certain settings [8,9], and is commonly accepted as being a sensible benchmark. An acceptance rate that is close to zero implies inefficient mixing, while an acceptance rate that is close to one implies the probability space is not efficiently explored. The algorithm proceeds as follows:

• For each component *c *of parameter *p*, assign a parameter-specific starting value to the corresponding proposal variance, σcp2
 MathType@MTEF@5@5@+=feaafiart1ev1aaatCvAUfKttLearuWrP9MDH5MBPbIqV92AaeXatLxBI9gBaebbnrfifHhDYfgasaacPC6xNi=xH8viVGI8Gi=hEeeu0xXdbba9frFj0xb9qqpG0dXdb9aspeI8k8fiI+fsY=rqGqVepae9pg0db9vqaiVgFr0xfr=xfr=xc9adbaqaaeGacaGaaiaabeqaaeqabiWaaaGcbaacciGae83Wdm3aa0baaSqaaiabdogaJjabdchaWbqaaiabikdaYaaaaaa@3174@

• Choose a sequence *δ *(*n*) → 0. We chose *δ *(*n*) = min(0.01, *n*^-1/2^)

• Start the MCMC simulation

• After the *n*^th ^batch of 50 iterations, calculate the acceptance ratio over the last batch

• If the acceptance ratio is less than the optimal value of 0.44, increase log(σcp2
 MathType@MTEF@5@5@+=feaafiart1ev1aaatCvAUfKttLearuWrP9MDH5MBPbIqV92AaeXatLxBI9gBaebbnrfifHhDYfgasaacPC6xNi=xH8viVGI8Gi=hEeeu0xXdbba9frFj0xb9qqpG0dXdb9aspeI8k8fiI+fsY=rqGqVepae9pg0db9vqaiVgFr0xfr=xfr=xc9adbaqaaeGacaGaaiaabeqaaeqabiWaaaGcbaacciGae83Wdm3aa0baaSqaaiabdogaJjabdchaWbqaaiabikdaYaaaaaa@3174@) by *δ *(*n*), else decrease it by *δ *(*n*)

The algorithm preserves ergodicity as long as each kernel has the right stationary distribution; the total variation distance between successive kernels tends to zero in probability; and the convergence time of each kernel is bounded in probability [6].

#### R package

The C++ component of BGX is compiled as a shared object which is loaded and executed automatically from within the R package [10]. BGX integrates standard Bioconductor classes such as AffyBatch to store raw microarray data and ExpressionSet to store processed gene expression measures. Users interested in running BGX programmatically from a shell script, for instance, or in a more memory-efficient manner, also have the choice to run a standalone binary version of the program.

## Results and Discussion

### Usage

The *bgx *package and its dependencies, *affy *and *gcrma*, may be installed automatically from the Bioconductor repository from an R shell. The package contains documentation and executable examples in a "vignette" file available using openVignette(). Users who wish to compile *bgx *from source will require the Boost C++ libraries [11] and the hgu95av2cdf Bioconductor package. The core functionality of the package is contained in the bgx function, which takes an AffyBatch object instantiated from one or more GeneChip CEL files as its first argument and returns an ExpressionSet object containing expression values for each gene and condition:

aData <- ReadAffy("chip1.CEL","chip2.CEL")

eset <- bgx(aData)

assayData(eset)$exprs # Returns expression values

assayData(eset)$se.exprs # Returns standard errors for expression values

Optional arguments include samplesets, which specifies the experimental design; genes, which specifies a subset of genes to analyse; burnin and iter, which specify the number of iterations for the burn-in and post burn-in phases of the algorithm respectively; probeAff, which specifies whether or not to use the probe affinity extension to the original BGX model, and adaptive, which specifies whether or not to use Metropolis-Within-Gibbs step adaptation. Full documentation for the bgx function is available by running help(bgx).

Although the point measures returned in the ExpressionSet object are useful, the distinctive power of the BGX method is that it provides samples from the full posterior distributions of the expression parameter, *μ*_*gc*_. These samples are, by default, saved in directories named run.1, run.2, *etc*. in R's current working directory, although this may be overridden with the rundir argument. They may be read into R in order to analyse the results of a simulation as follows:

bgxOutput <- readOutput.bgx("run.1")

**Figure 1:**
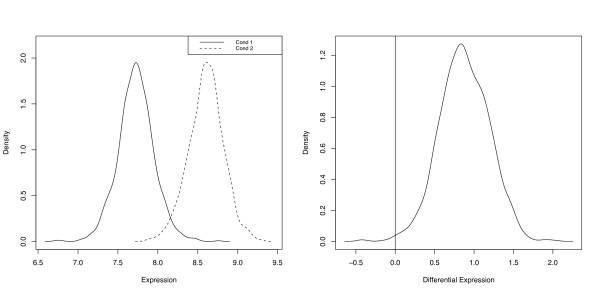
**Expression and differential expression densities**. plotExpressionDensity plots the density of    the posterior distribution of a given gene under each condition    (left). plotDEDensity plots the density of the difference in    the posterior distributions of a given gene between two conditions    (right).

**Figure 2:**
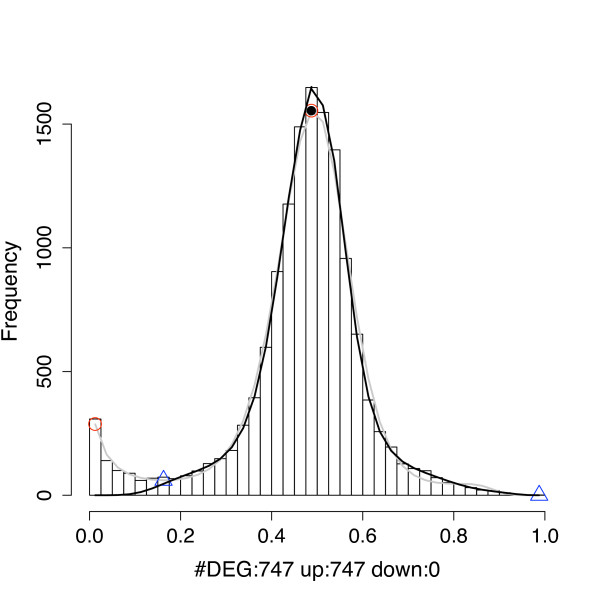
**Estimating the number of differentially expressed genes**. plotDEHistogram plots a histogram of *P*(*μ*_*g*2 _- *μ*_*g*1 _< 0), which is used to estimate the number of up and down-regulated genes between two conditions using a routine incorporated in the package.

The bgxOutput object is assigned to a list containing values from the full posterior distributions of *μ*_*gc *_(bgxOutput$mu), their expected values (bgxOutput$muave) and the gene names (bgxOutput$geneNames). This object can be passed to a number of functions to analyse the results. In particular, this object provides a direct measure of the variance of gene expression, a quantity which is only sometimes available when fitting robust linear models (e.g. using the AffyPLM Bioconductor package). The difference in expression between two conditions, *μ*_*g*2 _- *μ*_*g*1_, may be visualised with the plotExpressionDensity and plotDEDensity functions (Figure 1). plotDEHistogram fits a spline to the histogram of *P*(*μ*_*g*2 _- *μ*_*g*1 _< 0) using Poisson regression, estimates the null distribution by spline fitting of the central part of the histogram, and uses the difference between the histogram fit and the null distribution to give a preliminary estimate of the number of differentially expressed genes (Figure 2) [2]. A more thorough approach to the problem of classifying genes by differential expression may be found in the BGmix Bioconductor package — an implementation of a fully Bayesian mixture model for differential expression – which can analyse BGX output directly (Lewin, Bochkina and Richardson: Fully Bayesian mixture model for differential gene expression: simulations and model checks, submitted). rankByDE returns a matrix that ranks genes by their standardised BGX differences between two conditions (see Equation (7)) and specifies each gene's name, index and differential expression measure. More information on each function is available via help(analysis.bgx).

For the purposes of this paper, BGX was run with the "gold standard" of 16 k burn-in iterations and 64 k sampling iterations. However, the recommended 8 k burn-in iterations and 16 k sampling iterations are sufficient to provide good estimates of *μ*_*g *_(Additional File 1). Under these settings, BGX takes approximately one hour per array on a standard 64-bit 3 GHz computer. Analyses of up to 100 arrays ought to "fit" in a computer equipped with 4 GB of memory. However, BGX may be run separately for each condition, and the output subsequently combined in R by passing multiple output directories to the readOutput.bgx function. Since *φ*, the only parameter that is shared between conditions, is very stable for a given type of array, the impact on the output of running BGX separately on each condition is negligible.

### Estimation of non-specific hybridisation

**Figure 3:**
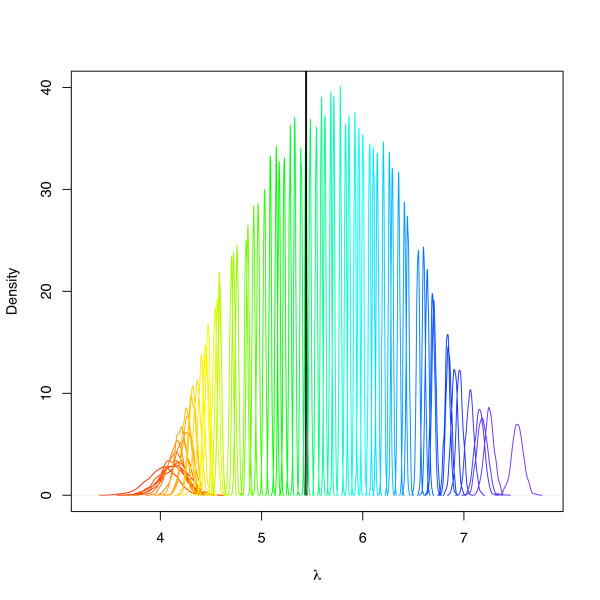
**Distributions of *λ *for each probe affinity category**. Colour-coded density plot of the λcrα(g,j)
 MathType@MTEF@5@5@+=feaafiart1ev1aaatCvAUfKttLearuWrP9MDH5MBPbIqV92AaeXatLxBI9gBaebbnrfifHhDYfgasaacPC6xNi=xH8viVGI8Gi=hEeeu0xXdbba9frFj0xb9qqpG0dXdb9aspeI8k8fiI+fsY=rqGqVepae9pg0db9vqaiVgFr0xfr=xfr=xc9adbaqaaeGacaGaaiaabeqaaeqabiWaaaGcbaacciGae83UdW2aa0baaSqaaiabdogaJjabdkhaYbqaaiab=f7aHjabcIcaOiabdEgaNjabcYcaSiabdQgaQjabcMcaPaaaaaa@3757@ parameter of an analysis of the Golden Spike data set. As probe affinity categories increase, the distributions shift from left to right. The black density line is the *λ*_*cr *_distribution from the original BGX model and illustrates the discriminatory power of the probe affinity extension.

The GCBGX model groups MM probes into categories that have similar probe affinities based on their oligonucleotide content. Instead of using a single array-specific parameter, *λ*_*cr *_(Equation (4)), we associate an appropriate λcrα(g,j)
 MathType@MTEF@5@5@+=feaafiart1ev1aaatCvAUfKttLearuWrP9MDH5MBPbIqV92AaeXatLxBI9gBaebbnrfifHhDYfgasaacPC6xNi=xH8viVGI8Gi=hEeeu0xXdbba9frFj0xb9qqpG0dXdb9aspeI8k8fiI+fsY=rqGqVepae9pg0db9vqaiVgFr0xfr=xfr=xc9adbaqaaeGacaGaaiaabeqaaeqabiWaaaGcbaacciGae83UdW2aa0baaSqaaiabdogaJjabdkhaYbqaaiab=f7aHjabcIcaOiabdEgaNjabcYcaSiabdQgaQjabcMcaPaaaaaa@3757@ component with each probe (Equation (5)), which should correlate positively with probe affinity categories. Figure 3 shows a colour-coded density plot of the λcrα(g,j)
 MathType@MTEF@5@5@+=feaafiart1ev1aaatCvAUfKttLearuWrP9MDH5MBPbIqV92AaeXatLxBI9gBaebbnrfifHhDYfgasaacPC6xNi=xH8viVGI8Gi=hEeeu0xXdbba9frFj0xb9qqpG0dXdb9aspeI8k8fiI+fsY=rqGqVepae9pg0db9vqaiVgFr0xfr=xfr=xc9adbaqaaeGacaGaaiaabeqaaeqabiWaaaGcbaacciGae83UdW2aa0baaSqaaiabdogaJjabdkhaYbqaaiab=f7aHjabcIcaOiabdEgaNjabcYcaSiabdQgaQjabcMcaPaaaaaa@3757@ parameter obtained in an analysis of the Golden Spike data [12]. As probe affinity categories increase, the distributions shift from left to right. The black density line corresponds to the *λ*_*cr *_distribution in the original BGX model and highlights the discriminatory power of the probe affinity extension.

**Figure 4:**
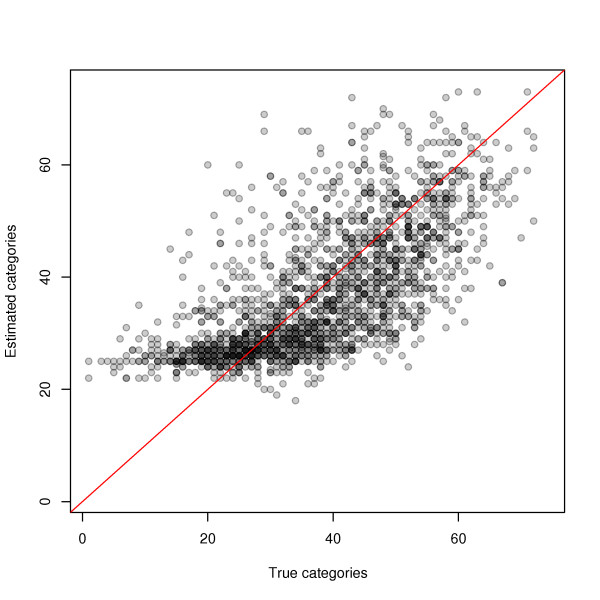
**Estimating the affinity of probes with unknown sequence**. Sequence information was artificially removed from one out ever 100 genes from the Golden Spike data set and the corresponding probes' affinity categories were estimated from the data. There is a positive correlation between estimated and true categories, particularly for high-affinity probes.

Probes with unknown sequences have their affinity categories estimated from the data. In order to check the effectiveness of this approach, we performed cross-validation on one out of every 100 genes from the Golden Spike data set, and compared the median estimated category to its true value. Figure 4 shows a positive correlation between estimated and true categories, particularly for high-affinity probes.

### Performance of adaptive MCMC

BGX is a computationally intensive program and it is therefore desirable to use an MCMC algorithm that mixes efficiently. One quantity of interest in this respect is the integrated autocorrelation time (IACT), which inflates the variance of the sample mean, X¯
 MathType@MTEF@5@5@+=feaafiart1ev1aaatCvAUfKttLearuWrP9MDH5MBPbIqV92AaeXatLxBI9gBaebbnrfifHhDYfgasaacPC6xNi=xH8viVGI8Gi=hEeeu0xXdbba9frFj0xb9qqpG0dXdb9aspeI8k8fiI+fsY=rqGqVepae9pg0db9vqaiVgFr0xfr=xfr=xc9adbaqaaeGacaGaaiaabeqaaeqabiWaaaGcbaGafmiwaGLbaebaaaa@2D24@[13]. To be precise, the variance of the sample mean may be expressed as follows [14]:

var(X¯)=E[S2(X)n]⋅n−1(n/a(X))−1
 MathType@MTEF@5@5@+=feaafiart1ev1aaatCvAUfKttLearuWrP9MDH5MBPbIqV92AaeXatLxBI9gBaebbnrfifHhDYfgasaacPC6xNi=xI8qiVKYPFjYdHaVhbbf9v8qqaqFr0xc9vqFj0dXdbba91qpepeI8k8fiI+fsY=rqGqVepae9pg0db9vqaiVgFr0xfr=xfr=xc9adbaqaaeGacaGaaiaabeqaaeqabiWaaaGcbaacbiGae8NDayNae8xyaeMae8NCaiNaeiikaGIafmiwaGLbaebacqGGPaqkcqGH9aqpcqWGfbqrdaWadaqcfayaamaalaaabaGaem4uam1aaWbaaeqabaGaeGOmaidaaiabcIcaOiabdIfayjabcMcaPaqaaiabd6gaUbaaaOGaay5waiaaw2faaiabgwSixNqbaoaalaaabaGaemOBa4MaeyOeI0IaeGymaedabaGaeiikaGIaemOBa4Maei4la8IaemyyaeMaeiikaGIaemiwaGLaeiykaKIaeiykaKIaeyOeI0IaeGymaedaaaaa@4ED0@

where *X *is an autocorrelated sample of size *n *with empirical mean X¯
 MathType@MTEF@5@5@+=feaafiart1ev1aaatCvAUfKttLearuWrP9MDH5MBPbIqV92AaeXatLxBI9gBaebbnrfifHhDYfgasaacPC6xNi=xH8viVGI8Gi=hEeeu0xXdbba9frFj0xb9qqpG0dXdb9aspeI8k8fiI+fsY=rqGqVepae9pg0db9vqaiVgFr0xfr=xfr=xc9adbaqaaeGacaGaaiaabeqaaeqabiWaaaGcbaGafmiwaGLbaebaaaa@2D24@, S2(X)=1n−1∑i(Xi−X¯)2
 MathType@MTEF@5@5@+=feaafiart1ev1aaatCvAUfKttLearuWrP9MDH5MBPbIqV92AaeXatLxBI9gBaebbnrfifHhDYfgasaacPC6xNi=xH8viVGI8Gi=hEeeu0xXdbba9frFj0xb9qqpG0dXdb9aspeI8k8fiI+fsY=rqGqVepae9pg0db9vqaiVgFr0xfr=xfr=xc9adbaqaaeGacaGaaiaabeqaaeqabiWaaaGcbaGaem4uam1aaWbaaSqabeaacqaIYaGmaaGccqGGOaakcqWGybawcqGGPaqkcqGH9aqpjuaGdaWcaaqaaiabigdaXaqaaiabd6gaUjabgkHiTiabigdaXaaakmaaqababaGaeiikaGIaemiwaG1aaSbaaSqaaiabdMgaPbqabaGccqGHsislcuWGybawgaqeaiabcMcaPmaaCaaaleqabaGaeGOmaidaaaqaaiabdMgaPbqab0GaeyyeIuoaaaa@4202@, and *a*(*X*) is the integrated autocorrelation time (IACT), defined as

a(X)=[1+2∑j=1n−1(1−jn)Pj]
 MathType@MTEF@5@5@+=feaafiart1ev1aaatCvAUfKttLearuWrP9MDH5MBPbIqV92AaeXatLxBI9gBaebbnrfifHhDYfgasaacPC6xNi=xI8qiVKYPFjYdHaVhbbf9v8qqaqFr0xc9vqFj0dXdbba91qpepeI8k8fiI+fsY=rqGqVepae9pg0db9vqaiVgFr0xfr=xfr=xc9adbaqaaeGacaGaaiaabeqaaeqabiWaaaGcbaGaemyyaeMaeiikaGIaemiwaGLaeiykaKIaeyypa0ZaamWaaeaacqaIXaqmcqGHRaWkcqaIYaGmdaaeWbqaamaabmaabaGaeGymaeJaeyOeI0scfa4aaSaaaeaacqWGQbGAaeaacqWGUbGBaaaakiaawIcacaGLPaaacqWGqbaudaWgaaWcbaGaemOAaOgabeaaaeaacqWGQbGAcqGH9aqpcqaIXaqmaeaacqWGUbGBcqGHsislcqaIXaqma0GaeyyeIuoaaOGaay5waiaaw2faaaaa@486B@

where

Pj=cov(Xi,Xi+j)var(Xi)var(Xi+j).
 MathType@MTEF@5@5@+=feaafiart1ev1aaatCvAUfKttLearuWrP9MDH5MBPbIqV92AaeXatLxBI9gBaebbnrfifHhDYfgasaacPC6xNi=xI8qiVKYPFjYdHaVhbbf9v8qqaqFr0xc9vqFj0dXdbba91qpepeI8k8fiI+fsY=rqGqVepae9pg0db9vqaiVgFr0xfr=xfr=xc9adbaqaaeGacaGaaiaabeqaaeqabiWaaaGcbaGaemiuaa1aaSbaaSqaaiabdQgaQbqabaGccqGH9aqpjuaGdaWcaaqaaiabdogaJjabd+gaVjabdAha2jabcIcaOiabdIfaynaaBaaabaGaemyAaKgabeaacqGGSaalcqWGybawdaWgaaqaaiabdMgaPjabgUcaRiabdQgaQbqabaGaeiykaKcabaWaaOaaaeaacqWG2bGDcqWGHbqycqWGYbGCcqGGOaakcqWGybawdaWgaaqaaiabdMgaPbqabaGaeiykaKIaemODayNaemyyaeMaemOCaiNaeiikaGIaemiwaG1aaSbaaeaacqWGPbqAcqGHRaWkcqWGQbGAaeqaaiabcMcaPaqabaaaaOGaeiOla4caaa@534C@

Evidently, if the sample is not autocorrelated, then *P*_*j *_= 0 for all *j*, *a*(*X*) collapses to 1 and *var*(X¯
 MathType@MTEF@5@5@+=feaafiart1ev1aaatCvAUfKttLearuWrP9MDH5MBPbIqV92AaeXatLxBI9gBaebbnrfifHhDYfgasaacPC6xNi=xH8viVGI8Gi=hEeeu0xXdbba9frFj0xb9qqpG0dXdb9aspeI8k8fiI+fsY=rqGqVepae9pg0db9vqaiVgFr0xfr=xfr=xc9adbaqaaeGacaGaaiaabeqaaeqabiWaaaGcbaGafmiwaGLbaebaaaa@2D24@) becomes equal to the familiar expression for an IID sample, E [*S*^2^(*X*)/*n*]. From (6), the IACT of a chain relates positively with the variability of its mean, and thus highly autocorrelated chains lead to poor estimates of our gene expression measure.

**Figure 5:**
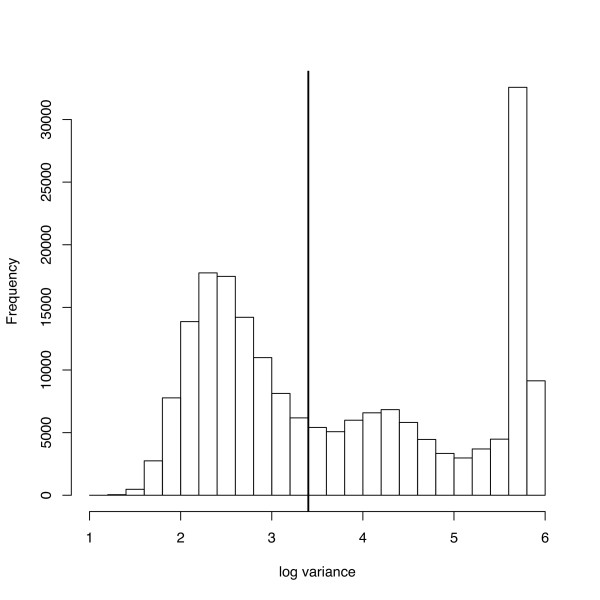
**Adapting the size of MCMC proposal steps**. When the adaptive MCMC algorithm is used, the variance of the Metropolis-Hastings proposal step is adapted independently for each *S *component. The plot shows a histogram of the optimal log variance for *S *proposals and the fixed step size used in the non-adaptive version overlaid in black, highlighting that a wide range of proposal variances are needed.

**Figure 6:**
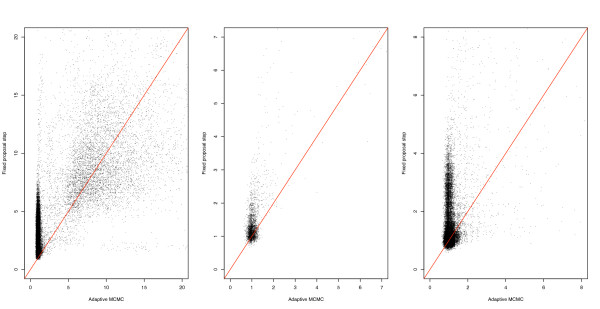
**Decreasing the IACT of expressed genes**. The plots show a dramatic reduction in the IACT of the *S *parameter and a milder improvement on the *μ *parameter of expressed genes (left & centre). A similar improvement was observed for the IACT of the *H *parameter, for all genes (right).

One way of improving our estimates is to increase the number of iterations while maintaining a fixed subsample size. This translates to subsamples being further apart on the original chain and therefore less correlated. It is faster and more attractive, however, to use an adaptive algorithm that explores the probability space more efficiently. Using the Golden Spike data set [12] for our investigation, we found that the adaptive method led to a range of optimal proposal magnitudes for the Metropolis-Hastings parameters. Figure 5 illustrates this with a histogram of the optimal log variance for *S *proposals on one array and the original fixed step size overlaid in black. Figure 6 (left & centre) shows a dramatic reduction in the IACT of the *S *parameters and a milder improvement on the *μ*_*g *_parameters of expressed genes. A similar improvement was observed for the IACT of the *H *parameters, this time for all genes (Figure 6 right).

Differential expression can be quantified by the standardised BGX differences between two conditions:

zg=d¯gvar(d¯g)
 MathType@MTEF@5@5@+=feaafiart1ev1aaatCvAUfKttLearuWrP9MDH5MBPbIqV92AaeXatLxBI9gBaebbnrfifHhDYfgasaacPC6xNi=xI8qiVKYPFjYdHaVhbbf9v8qqaqFr0xc9vqFj0dXdbba91qpepeI8k8fiI+fsY=rqGqVepae9pg0db9vqaiVgFr0xfr=xfr=xc9adbaqaaeGacaGaaiaabeqaaeqabiWaaaGcbaGaemOEaO3aaSbaaSqaaiabdEgaNbqabaGccqGH9aqpdaWcaaqaaiqbdsgaKzaaraWaaSbaaSqaaiabdEgaNbqabaaakeaadaGcaaqaaiabdAha2jabdggaHjabdkhaYjabcIcaOiqbdsgaKzaaraWaaSbaaSqaaiabdEgaNbqabaGccqGGPaqkaSqabaaaaaaa@3C27@

where, from (6), *var*(d¯g
 MathType@MTEF@5@5@+=feaafiart1ev1aaatCvAUfKttLearuWrP9MDH5MBPbIqV92AaeXatLxBI9gBaebbnrfifHhDYfgasaacPC6xNi=xH8viVGI8Gi=hEeeu0xXdbba9frFj0xb9qqpG0dXdb9aspeI8k8fiI+fsY=rqGqVepae9pg0db9vqaiVgFr0xfr=xfr=xc9adbaqaaeGacaGaaiaabeqaaeqabiWaaaGcbaGafmizaqMbaebadaWgaaWcbaGaem4zaCgabeaaaaa@2EBF@) is estimated by

var(d¯g)_=S2(dg)n⋅n−1(n/a(dg)_−1,
 MathType@MTEF@5@5@+=feaafiart1ev1aaatCvAUfKttLearuWrP9MDH5MBPbIqV92AaeXatLxBI9gBaebbnrfifHhDYfgasaacPC6xNi=xI8qiVKYPFjYdHaVhbbf9v8qqaqFr0xc9vqFj0dXdbba91qpepeI8k8fiI+fsY=rqGqVepae9pg0db9vqaiVgFr0xfr=xfr=xc9adbaqaaeGacaGaaiaabeqaaeqabiWaaaGcbaWaaecaaeaacqWG2bGDcqWGHbqycqWGYbGCcqGGOaakcuWGKbazgaqeamaaBaaaleaacqWGNbWzaeqaaOGaeiykaKcacaGLcmaacqGH9aqpjuaGdaWcaaqaaiabdofatnaaCaaabeqaaiabikdaYaaacqGGOaakcqWGKbazdaWgaaqaaiabdEgaNbqabaGaeiykaKcabaGaemOBa4gaaOGaeyyXICDcfa4aaSaaaeaacqWGUbGBcqGHsislcqaIXaqmaeaacqGGOaakcqWGUbGBcqGGVaWldaqiaaqaaiabdggaHjabcIcaOiabdsgaKnaaBaaabaGaem4zaCgabeaacqGGPaqkaiaawkWaaiabgkHiTiabigdaXaaakiabcYcaSaaa@5225@

*dg *= *μ*_*g*2 _- *μ*_*g*1_, *g *= 1 ..., *G*, that is, samples from the posterior distribution of the difference in the BGX expression measure for each gene, and a(dg)_
 MathType@MTEF@5@5@+=feaafiart1ev1aaatCvAUfKttLearuWrP9MDH5MBPbIqV92AaeXatLxBI9gBaebbnrfifHhDYfgasaacPC6xNi=xH8viVGI8Gi=hEeeu0xXdbba9frFj0xb9qqpG0dXdb9aspeI8k8fiI+fsY=rqGqVepae9pg0db9vqaiVgFr0xfr=xfr=xc9adbaqaaeGacaGaaiaabeqaaeqabiWaaaGcbaqcfa4aaecaaeaacqWGHbqycqGGOaakcqWGKbazdaWgaaqaaiabdEgaNbqabaGaeiykaKcacaGLcmaaaaa@32E9@, the estimate of the Monte Carlo standard error, is calculated using Sokal's adaptive truncated periodogram estimator [?]. Our z-score differs from the measure used in [2] by a factor of ((n/a(dg)_−1)/(n−1)
 MathType@MTEF@5@5@+=feaafiart1ev1aaatCvAUfKttLearuWrP9MDH5MBPbIqV92AaeXatLxBI9gBaebbnrfifHhDYfgasaacPC6xNi=xH8viVGI8Gi=hEeeu0xXdbba9frFj0xb9qqpG0dXdb9aspeI8k8fiI+fsY=rqGqVepae9pg0db9vqaiVgFr0xfr=xfr=xc9adbaqaaeGacaGaaiaabeqaaeqabiWaaaGcbaqcfa4aaOaaaeaacqGGOaakcqGGOaakcqWGUbGBcqGGVaWldaqiaaqaaiabdggaHjabcIcaOiabdsgaKnaaBaaabaGaem4zaCgabeaacqGGPaqkaiaawkWaaiabgkHiTiabigdaXiabcMcaPiabc+caViabcIcaOiabd6gaUjabgkHiTiabigdaXiabcMcaPaqabaaaaa@3F85@, which takes into account the autocorrelation structure of the sequence of values generated by the algorithm. Since the adaptive MCMC algorithm has the effect of decreasing *a*(*d*_*g*_) for expressed genes while keeping it approximately constant for non-expressed genes, it leads to an increase in the ranking of expressed genes and consequently in BGX's capacity to detect differential expression.

### Performance on spike-in datasets

We illustrate the performance of *bgx *by presenting detailed results from analyses of arrays from the Affymetrix Latin Square data [15] and the Golden Spike data set [12].

#### Latin Square data

Affymetrix published two data sets for assessing the performance of expression algorithms on their microarrays. The HGU95A data set consists of 16 genes spiked in at known concentrations ranging from 0 to 1024 pM and arrayed in a Latin Square format. We considered 16 instead of the original 14 genes described by Affymetrix because we included two extra spike-ins, 546_at and 33818_at, as reported in [16]. We used two replicates and 14 unique concentration configurations labelled A to M and Q. 2716 of the probes in this data set had no sequence information and therefore their probe affinity categories were estimated from the data as part of the model. The HGU133A data set consists of 64 genes spiked in at known concentrations ranging from 0 to 512 pM. We considered 64 instead of the original 42 genes described by Affymetrix because we included 22 extra spike-ins, as reported in [17]. We used all 3 replicates for each of the 14 concentration groups.

**Figure 7:**
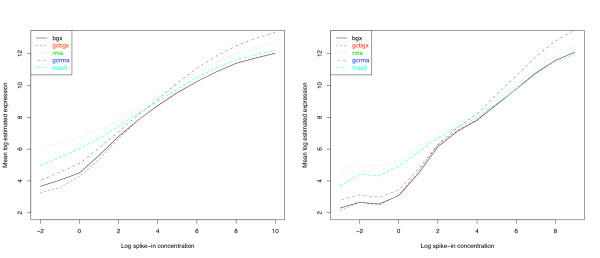
**Performance on the Latin Square data**. Log concentration vs. mean log estimated expression    of spike-in genes in the HGU95A (left) and HGU133A (right) Latin    Square data sets. There is an increased ability to detect    concentration changes at levels lower than 4 pM in the HGU95A data set    using GCBGX instead of BGX. Both BGX and GCBGX are more sensitive to    changes within the low range than RMA or GCRMA and more sensitive to    changes between 0.5 pM and 16 pM than MAS5. The MAS5 concentration    line was shifted down by 2.5 units to facilitate the comparison of its    gradient with those of the other lines.

The data from these experiments were analysed using BGX, GCBGX, RMA [18], GCRMA [4] and MAS5 [?], and the average expression for each concentration level was recorded. Figure 7 (left) shows a steeper gradient at levels lower than 4 pM in the HGU95A data set using GCBGX instead of BGX, pointing to an increased ability to detect concentration changes. For both data sets, BGX and GCBGX are more sensitive to changes within the low range than RMA, GCRMA or MAS5 (Figure 7 left & right).

#### Golden Spike data set

The Golden Spike data set consists of six DrosGenome1 GeneChips, with three technical replicates from two conditions: C and S. There are 14010 probe sets in each array representing 14010 genes. 2535 of these are expressed equally under both conditions while 1331 genes are up-regulated in S relative to C. The data is highly valuable for comparing chip analysis methods because it is fully controlled and contains very realistic noise. Due to the asymmetry of the spike-ins, a normalisation of the posterior distributions similar to that advocated in [12] was carried out by fitting a loess curve to the MA plot [19] of the posterior mean values of *μ*_*gc *_for the non-differentially expressed genes, predicting a curve from the fit for all genes, and subtracting the curve from the posterior distributions of the differences in expression. The RMA, GCRMA and MAS5 expression measures were similarly adjusted using loess normalisation at the probeset level instead of the default quantile normalisation at the probe level.

**Figure 8:**
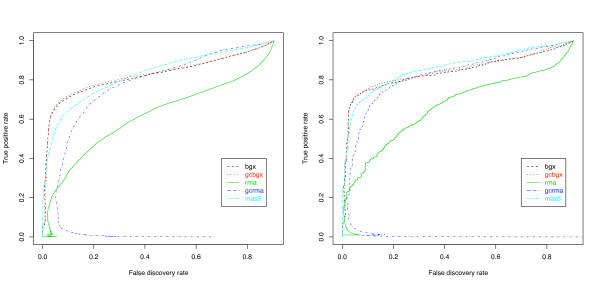
**Performance on the Golden Spike data**. Nine single-array comparisons (left) and three-   replicate comparisons (right) between condition C and S were performed    using BGX, GCBGX, RMA, GCRMA and MAS5. The ROC curves show that GCBGX    has a small advantage over BGX and that both models perform well. For    false discovery rates below 20%, BGX and GCBGX outperform RMA, GCRMA    and MAS5.

Receiver operating characteristic (ROC) curves depict the observed false discovery rate vs. the true positive rate as the cut-off of a ranked gene list is varied. Figure 8 (left) shows average ROC curves for the nine single-array comparisons between condition C and condition S while Figure 8 (right) shows ROC curves for three-replicate comparisons. GCBGX has a small advantage over BGX and both models perform well. In the single-array comparisons, BGX and GCBGX outperform RMA, GCRMA and MAS5 for false discovery rates below 30%. The number of differentially expressed genes (DEGs) were estimated by running plotDEHistogram on the output of the nine comparisons involving one array from condition C versus one array from condition S. The number of genes detected as up-regulated with GCBGX ranged from 681 to 883 (mean 783) and for BGX from 560 to 867 (mean 742). Both methods produced an average true positive rate across the nine comparisons of over 97% for up-regulated genes. In the three-replicate comparisons, BGX and GCBGX outperform RMA, GCRMA and MAS5 for false discovery rates below 20%. An analysis of a three-replicate comparison yielded 1002 and 958 DEGs with 96.6% and 95.7% true positive rates using GCBGX and BGX respectively.

## Conclusion

BGX is a new Bioconductor R package for analysing 3' Affymetrix GeneChips. BGX implements a fully integrated Bayesian hierarchical model with the option to take into account sequence-dependent probe affinities. BGX uses a novel adaptive MCMC algorithm that improves the efficiency with which the posterior distributions of parameters are sampled from. BGX compares favourably to RMA and GCRMA at detecting differential expression, particularly at low concentration levels.

## Availability and requirements

**Project name: **BGX

**Project homepage: **

**Operating systems: **Platform independent

**Programming language: **C++, R

**Other requirements: **R, Bioconductor

**License: **GNU GPL

**Any restrictions to use by non-academics: **No

## Authors' contributions

ET implemented and tested the code and developed the adaptive MCMC. AKH and SR extended the BGX model to take into account probe affinity effects. NB and ET modelled and implemented probe affinity estimation of probes with missing sequences. ET, AKH, NB and SR provided comments and discussion and wrote the paper. All authors read and approved the final manuscript.

## Supplementary Material

Additional file 1 **Varying the number of sampling iterations**. Plot of mean posterior values of *μ*_*g *_obtained using a sampling length of 64 k iterations versus those obtained using sampling lengths of 16 k, 32 k and 64 k using an alternative seed for the pseudo random number generator. The values for the 32 k and 64 k comparisons are shifted upwards for clarity. Runs of more than 16 k confer a small increase in stability of estimation for non-expressed genes. At 32 k, estimates are as stable as between two 64 k runs using different seeds. Click here for file

Additional file 2 **R script to reproduce plots**. R script to reproduce the plots in this paper. Click here for file
