# Supplementary figures and images for: BGX: a Bioconductor package for the Bayesian integrated analysis of Affymetrix GeneChips
Source: BMC Bioinformatics. 2007 Nov 12;8:439. doi: 10.1186/1471-2105-8-439 (PMC2216047; doi:10.1186/1471-2105-8-439)

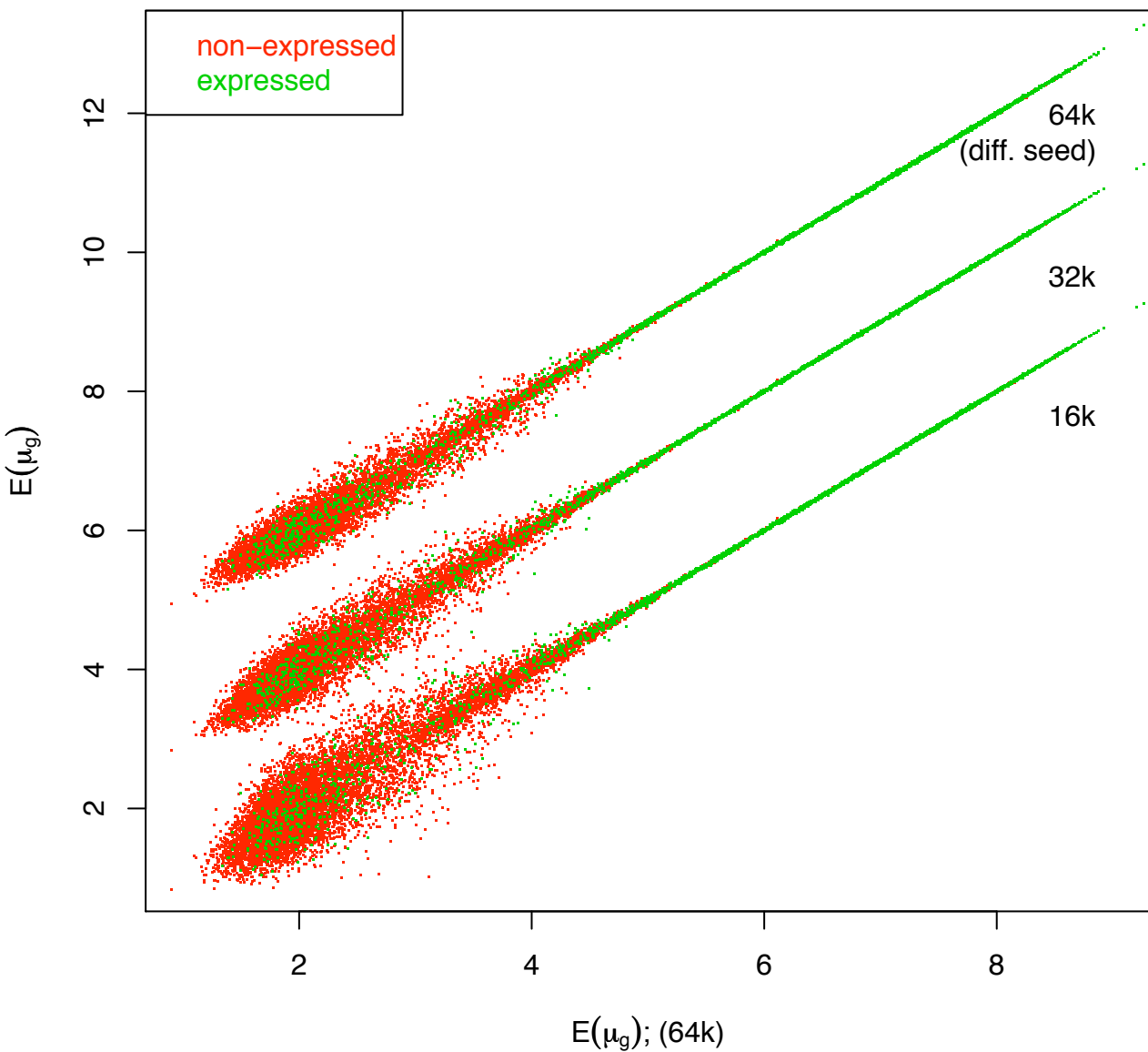

Supplement: Additional file 1 — Varying the number of sampling iterations. Plot of mean posterior values of μg obtained using a sampling length of 64 k iterations versus those obtained using sampling lengths of 16 k, 32 k and 64 k using an alternative seed for the pseudo random number generator. The values for the 32 k and 64 k comparisons are shifted upwards for clarity. Runs of more than 16 k confer a small increase in stability of estimation for non-expressed genes. At 32 k, estimates are as stable as between two 64 k runs using different seeds. [file 1471-2105-8-439-S1.pdf]
